# Neurologic Manifestations Associated with Parvovirus B19 Epidemic, Madrid, Spain, 2024

**DOI:** 10.3201/eid3108.250278

**Published:** 2025-08

**Authors:** Cristina Veintimilla, Pilar Catalán, Agustín Estévez, Roberto Alonso, Maricela Valerio, Patricia Muñoz

**Affiliations:** Clinical Microbiology and Infectious Diseases, Hospital General Universitario Gregorio Marañón, Madrid, Spain (C. Veintimilla, P. Catalán, A. Estévez, R. Alonso, M. Valerio, P. Muñoz); Instituto de Investigación Sanitaria Gregorio Marañón, Madrid (C. Veintimilla, P. Catalán, A. Estévez, R. Alonso, M. Valerio, P. Muñoz); School of Medicine, Universidad Complutense de Madrid, Madrid (P. Catalán, R. Alonso, M. Valerio, P. Muñoz); Centro de Investigación Biomédica en Red de Enfermedades Respiratorias, Madrid (P. Muñoz)

**Keywords:** parvovirus B19, viruses, central nervous system viral infections, infections, parvovirus, Spain, meningitis/encephalitis

## Abstract

A reemergence of parvovirus B19 infections in Spain in early 2024 prompted a 10-year review of the virus at a tertiary center. We identified 8 case-patients with neurologic manifestations who had parvovirus B19 in cerebrospinal fluid. Early recognition and management of parvovirus B19–associated neurologic conditions will help yield favorable outcomes.

Parvovirus B19 is a small, nonenveloped DNA virus that belongs to the Parvoviridae family. Parvovirus B19 infection generally manifests in paucisymptomatic or mild forms (*1*,*2*). However, severe symptoms, including chronic anemia, pancytopenia, and transient aplastic crisis, have been observed in at-risk populations, such as pregnant women, immunocompromised patients, and persons with chronic hematologic diseases. Neurologic manifestations of parvovirus B19 are rarely documented, underscoring the necessity for further epidemiologic and clinical investigation (*2*,*3*).

Community parvovirus B19 outbreaks typically peak in winter and spring and last an average of 3–6 months (*4*). Because parvovirus B19 is a nonnotifiable disease, testing practices vary between countries. Pregnant women and immunocompromised patients represent special cases for which testing is common (*1*).

Since March 2024, many countries in Europe, including Spain, have reported increased detection of parvovirus B19 (*1*). We observed a similar trend at Hospital Gregorio Marañón in Madrid, Spain, and we saw a larger peak of cases in the 2024 outbreak compared with the previous 10 years (Figure 1). After the reemergence of parvovirus B19, we observed the virus in cerebrospinal fluid (CSF) in some patients from our center. We investigated the clinical features and microbiological findings from case-patients with neurologic symptoms treated at Hospital Gregorio Marañón during 2014–2024.

**Figure 1:**
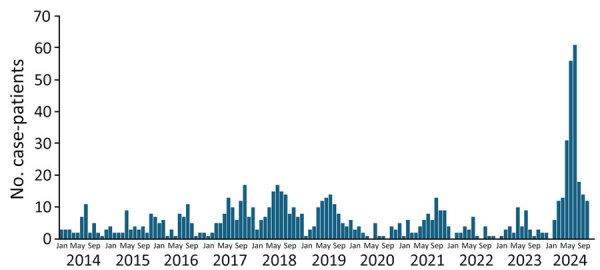
Parvovirus B19 case-patients confirmed by IgM during study of neurologic manifestations associated with parvovirus B19 outbreaks, Madrid, Spain, January 2014–October 2024. Numbers of case-patients are shown by month.

## The Study

We conducted a retrospective data extraction on CSF samples submitted to our laboratory for diagnostic workup of meningoencephalitis during January 2014–October 2024. We excluded bacterial and other viral causes of meningoencephalitis by using bacterial culture and molecular tests. We performed a review of demographic and clinical features on patients with CSF samples positive for parvovirus B19. We included serologic data when available by using an Alinity i System chemiluminescence assay (Abbott Laboratories, https://www.abbott.com). We performed molecular testing on CSF by using Allplex Meningitis V1-V2 Panel Assays multiplex PCR (Seegene, http://www.seegene.com) (Appendix). To ensure accuracy, we performed a second parvovirus B19–specific PCR on case-patients with virus detected in the central nervous system during 2024. For plasma samples, we used a specific target PCR (RealStar Parvovirus B19 PCR Kit; Altona Diagnostics, https://www.altona-diagnostics.com) and performed diagnostic testing procedures using the manufacturer’s specifications. Since 2019, our laboratory has been equipped with multiplex PCR for CSF; before 2019, we outsourced that test to the national reference laboratory in Madrid.

**Figure 2:**
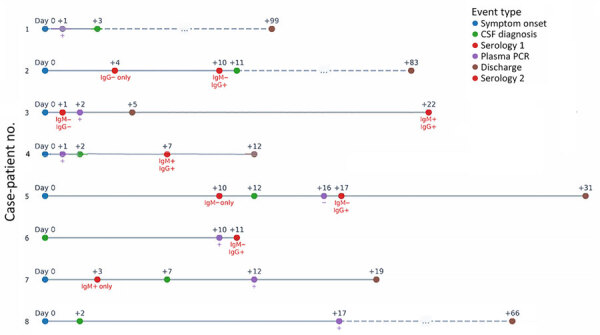
Clinical timelines for case-patients with neurologic manifestations associated with parvovirus B19 epidemic, Madrid, Spain, 2014–2024. CSF, cerebrospinal fluid.

During the January–October 2024 outbreak, we found 6 of 223 CSF samples tested were positive for parvovirus B19. An archival review of meningoencephalitis cases tested during 2014–2023 revealed 2 of 801 additional hospital patients with parvovirus B19 in CSF. The overall CSF parvovirus B19 positivity rate for the entire 10-year period was 0.8% (8 patients): 6 patients had no underlying immunosuppression, but the other 2 had a history of hematologic disease (Table; Figure 2). No cutaneous manifestations were observed. Central nervous system manifestations exhibited variability, and mental status changes were the most common. CSF analysis showed protein levels greater than the upper reference limit (i.e., >30 mg/dL) in all samples. Three patients received a 5-day course of intravenous immunoglobulin (IVIg), but no clinical benefit was observed between treated and untreated patients. All patients recovered completely without neurologic sequelae (range 3–21 days). Brain magnetic resonance imaging and computed tomography scans were unremarkable; the exception was case-patient 5, in whom a parenchymal lesion observed on the brain computed tomography scan was consistent with a high-grade lymphoproliferative disease. We performed cytology and confirmed lymphoproliferative disease (Appendix).

**Table:** Clinical and epidemiologic characteristics of parvovirus B19 in CSF of case-patients with neurologic manifestations associated with parvovirus B19 epidemic, Madrid, Spain, 2014–2024*

Characteristic	Case-patient no.							
	1	2	3	4	5	6	7	8
Age, y/sex	17/M	77/M	10/F	42/F	65/M	46/M	78/M	<1/M†
Diagnosis date	2014 Jan	2022 Aug	2024 May	2024 May	2024 May	2024 May	2024 Jun	2024 Aug 2
Location	IP	IP	IP	IP	IP	OP	IP	IP
Relevant medical history	Acute lymphocytic leukemia	Hepatic steatosis	Migraine with aura	Schizophrenia, migraine	Prostate cancer	BPDCN	COPD, hypertension	Premature birth
Neurologic symptoms	Mental status change	Mental status change, encephalopathy and delirium	Stroke	Visual hallucinations	Memory loss episodes	Persistent headache	Encephalopathy, mental status change	Poorly responsive and central seizures
Other symptoms and signs	Fever	None	Fever	Fever and arthralgias	None	None	Fever	None
Initial IgM/IgG, serum	−/−	NA/−	−/−‡	+/+	−/NA	−/+	+/NA	NA/NA
Hematologic findings	Anemia (8.6 g/dL), TCP (10,000/µL)	Anemia (7.7 g/dL), TCP (42,000/µL)	UN	Lymphopenia (300 cells/µL)	Anemia (8.6 g/dL)	UN	TCP (69,000/µL), leukopenia (2,600 cells/µL)	Anemia (9.5 g/dL), TCP (67,000/µL)
CSF								
Leukocytes	0	0	0	0	70	0	0	NA; RBCs in CSF
Protein level, mg/dL	87	42	40	39	129	36	49	136
Viral DNA	Positive	Positive	Positive	Positive	Positive	Positive	Positive	Positive
Plasma viral DNA	Positive	Positive	Positive	Positive	Negative	Negative	Positive	Positive
Treatment	IVIg	IVIg	2 doses acyclovir	1 dose acyclovir	No	IVIg	10 d acyclovir	No

*BPDCN, blastic plasmacytoid dendritic cell neoplasm; COPD, chronic obstructive pulmonary disease; CSF, cerebrospinal fluid; IP, inpatient; IVIg, intravenous immunoglobulin; NA, not available; OP, outpatient; RBCs, red blood cells (erythrocytes); TCP, thrombocytopenia. †Three-day-old infant. ‡Initial parvovirus B19 IgM and IgG were negative but seroconverted to 3 weeks later. §Upper normal limit 30 mg/dL.

This study lends support to the numerous reports indicating a rise of parvovirus B19 infection during the 2024 outbreak compared with previous years (*1**,**5*,*6*). However, the precise causes of the increase in case numbers remain unclear. Although a multitude of factors are likely implicated, reduced host immune response attributable to restrictions during the COVID-19 pandemic period warrants consideration. Moreover, the absolute increase in case numbers could result in a proportional rise of symptomatic cases, and some cases might have more severe clinical manifestations.

The association between parvovirus B19 infection and neurologic symptoms is poorly documented. Cases of parvovirus B19 infection in persons exhibiting neurologic manifestations are not distinguishable from cases of encephalitis caused by other viral agents. The proposed physio-pathologic mechanisms contributing to the development of neurologic manifestations during parvovirus B19 infection are complex and variable. Those mechanisms include direct viral toxicity, dysregulated immune responses with the release of cytokines in the CSF, immune complex deposition on endothelial cells, and intracellular accumulation of the toxic nonstructural 1 protein (*5*,*7*).

Some studies reported findings that support parvovirus B19 infection as a cause of neurologic manifestations. A comprehensive review identified 129 cases related to parvovirus B19 infection and neurologic symptoms; one third of the patients had a previous history of altered immunity (*2*). Another study found the most common parvovirus B19–associated neurologic manifestation was encephalitic syndromes (39%) (*8*). Rash was observed more frequently among immunocompetent patients than in those with altered immunity; in some cases, rash preceded the onset of other clinical findings (*2*,*8*). Arthralgia or arthritis symptoms were infrequent. CSF alterations did not show a clear pattern; the median leukocyte count was 9 cells/mL, and a slight increase in protein levels (51%) was the most notable finding. Up to 42% of cases had hematologic disturbances (*2*,*8*). 

In our case series, parvovirus B19 infection manifested in the absence of typical cutaneous manifestations. Among patients in our study, thrombocytopenia was the most prevalent hematologic disorder, and analysis of CSF samples revealed protein levels greater than the established threshold in all cases. In addition, most cases in our study were among adults without underlying immunocompromising diseases.

Parvovirus B19 is frequently underestimated in differential diagnosis schemes of meningoencephalitis. To accurately diagnose parvovirus B19 infection when neurologic involvement is observed, use of additional microbiological investigations is recommended, including serologic markers and virus DNA analysis in plasma and CSF.

In terms of management, steroids and IVIg have been identified as treatment options for clinical syndromes linked to parvovirus B19 infection. The choice to use IVIg is based on the assumption that it contains a substantial quantity of antibodies capable of neutralizing the virus. However, the precise mechanism of IVIg action remains uncertain (*2*,*8*). We did not observe any clinical differences between the patients who received IVIg and those who did not. The prognosis for neurologic manifestations associated with parvovirus B19 seems favorable; we saw a high rate of spontaneous recovery and an absence of sequelae. Nevertheless, long-term neurologic effects have been documented, including bradyphemia (slow speech), learning difficulties, and slurred speech, as well as more severe outcomes, such as mental and motor impairment and death (*2*,*8*). In this particular context, our findings are consistent with the available literature on parvovirus B19 infections with neurologic manifestations.

This study is limited by its retrospective design, single institution data collection, and small sample size of neurologic parvovirus B19 infections, which might bias the ability to draw definitive conclusions. Nevertheless, this study furnishes valuable information regarding viral determination in the CSF, thereby enabling the formulation of subsequent hypotheses and research initiatives.

## Conclusions

Our findings underscore the importance of incorporating parvovirus B19 into differential diagnoses of encephalitis, given its capacity to affect both immunocompetent and immunocompromised persons. Research is needed to elucidate the underlying mechanisms of parvovirus B19 to develop targeted treatments. Early recognition and appropriate management of parvovirus B19–associated neurologic conditions have the potential to yield favorable outcomes. 

Appendix Additional information for neurologic manifestations associated with parvovirus B19 epidemic, Madrid, Spain, 2024.
