# Supplementary material for: Neurologic Manifestations Associated with Parvovirus B19 Epidemic, Madrid, Spain, 2024
Source: Emerg Infect Dis. 2025 Aug;31(8):1621–4. doi: 10.3201/eid3108.250278 (PMC12309755; doi:10.3201/eid3108.250278)
Supplement: Appendix — Additional information for neurologic manifestations associated with parvovirus B19 epidemic, Madrid, Spain, 2024. [file 25-0278-Techapp-s1.pdf]

Article DOI: https://doi.org/10.3201/eid3108.250278

EID cannot ensure accessibility for supplementary materials supplied by authors. Readers who have difficulty accessing supplementary content should contact the authors for assistance.

Neurologic Manifestations Associated with Parvovirus B19 Epidemic, Madrid, Spain, 2024

Appendix

Clinical Description of Positive B19V Cases with Neurologic Manifestations

Case 1: A 18-yo male with a prior history of acute lymphoid leukemia, actively treated with chemotherapy. During an aplastic crisis, he was diagnosed with a reactivation of B19V confirmed in plasma and bone marrow. Three weeks later, he experienced mental status changes, visual hallucinations and delirium. B19V was confirmed in his CSF.

Case 2: A 77-yo male with an immunocompetent status. He was admitted to the ICU due to decrease in level of consciousness and the presence of delirium. The initial blood work-up revealed a bicytopenia, characterized by anemia (7.7 g/dL) and thrombocytopenia (42,000/µL). B19V was detected in his CSF, and intravenous immunoglobulins were administered as treatment.

Case 3: A 10-yo female with no underlying immune-related conditions. This patient was transferred to our center as a suspected stroke case, exhibiting paresthesia on the whole right-side of the body and aphasia that had lasted for four hours. The patient received 2 doses of acyclovir as an empirical treatment. During the initial admission period, she also presented febrile episodes. CT and MRI images did not reveal any pathological findings. Patient's bloodwork was within normal levels and initial B19V IgM and IgG were negative. CSF study detected B19V by PCR. Three weeks later, she seroconverted for B19V IgM and IgG antibodies.

Case 4: A 43-yo female, immunocompetent, reported a history of fever and arthralgias lasted for 1 week. During the admission process, she exhibited visual hallucinations. The blood work revealed lymphopenia (300 cells/µL) as a remarkable finding,

and all collected samples tested positive for B19V (serologies and blood and CSF PCR). The patient received 1 dose of acyclovir as an empiric treatment.

Case 5: A 66-year-old male with a medical history of localized prostate carcinoma, in remission since 2012. Due to amnesia, he attended the emergency department. Initial work-up showed negative B19V serologies and DNA in plasma. The diagnosis was confirmed on the basis of positive PCR findings on the CSF. No positive B19V results were obtained in other specimens for this patient.

Case 6: A 41-year-old male with a medical history of dendritic plasmocitoid cell neoplasm in 2022 and a hematopoietic stem cell transplant in 2023 presented to the emergency department with a subacute, persistent headache and blurred vision. CSF PCR revealed the presence of B19V. Serologic analysis revealed the presence of IgG antibodies, while DNA B19V testing was negative. No admission was necessary, but a course of intravenous immunoglobulins was administered.

Case 7: The patient is a 78-year-old male with no underlying immune-related health issues. Following a thorough evaluation, the patient was admitted with a suspected case of encephalitis. The symptoms include fever, behavioral changes and desorientation. The laboratory findings revealed thrombocytopenia (69,000/µL) and leukopenia (2,600 cells/µL), which led to a delay in lumbar puncture. The patient received acyclovir as a treatment, and 1 week later, a CSF PCR test revealed the presence of B19V. He had a positive DNA B19V in plasma and a positive IgM antibody result in serologic testing.

Case 8 was a 28-week-old premature who, 4 days after birth, presented with seizures, hypoactivity and signs of hypoperfusion. As sepsis was suspected, blood and urine cultures (no isolates were reported) were collected, and empirical antibiotics were administered. At day +6, the symptoms persisted and lumbar puncture was added to the diagnostic work-up. B19V was detected in the CSF. Plasma B19V PCR was performed with a positive result.

Supplemental Materials

As part of our daily routine protocol for the diagnosis of CSF viral infections, we employ a multiplex one-step real-time RT-PCR test (Seegene) which identify 12 viruses in two different panels:

- Allplex Meningitis V1 panel: *Cytomegalovirus* (CMV), Epstein-Barr virus (EBV), Herpes simplex virus type 1 (HSV1), Herpes simplex virus type 2 (HSV2), Human herpes virus 6 (HHV 6), Human herpes virus 7 (HHV 7), Varicella-zoster virus (VZV).

- Allplex Meningitis V2 panel: Adenovirus (AdV), Enterovirus (HEV), Human parechovirus (HPeV), Mumps virus (MV), Parvovirus B19 (B19V).

For plasma samples we used a specific target B19V RT-PCR (RealStar Parvovirus B19 PCR Kit, Altona Diagnostics).
